# Supplementary figures and images for: Arf1-dependent LRBA recruitment to Rab4 endosomes is required for endolysosome homeostasis
Source: J Cell Biol. 2024 Sep 26;223(11):e202401167. doi: 10.1083/jcb.202401167 (PMC11449124; doi:10.1083/jcb.202401167)

# Source Data Figure 1

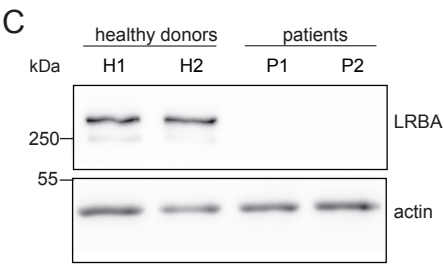

Source blots

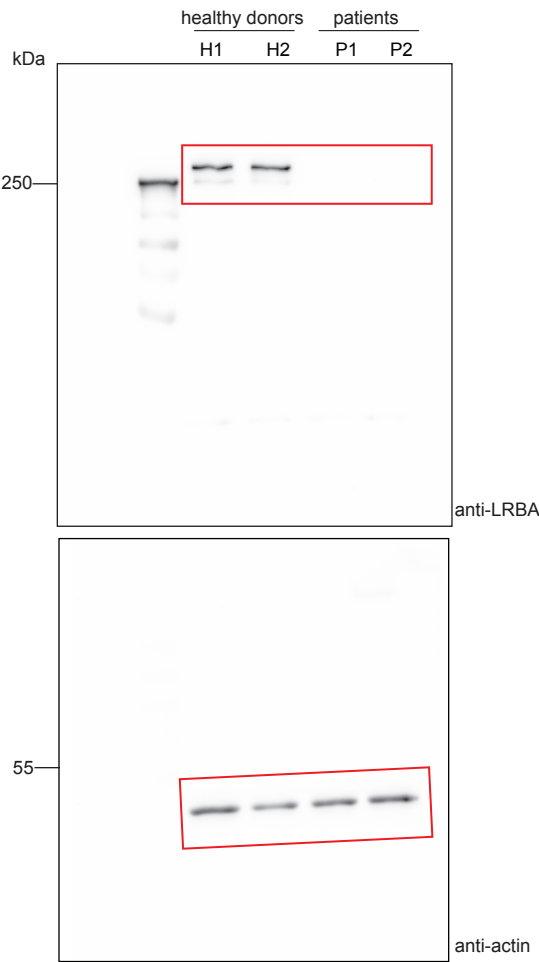

Supplement: SourceData F1 — is the source file for Fig. 1. [file JCB_202401167_SourceDataF1.pdf]

Source Data Figure4

E

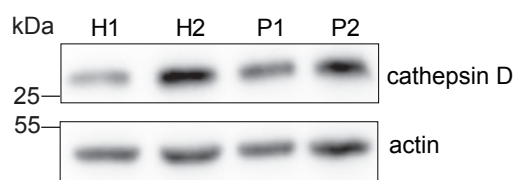

Source blots

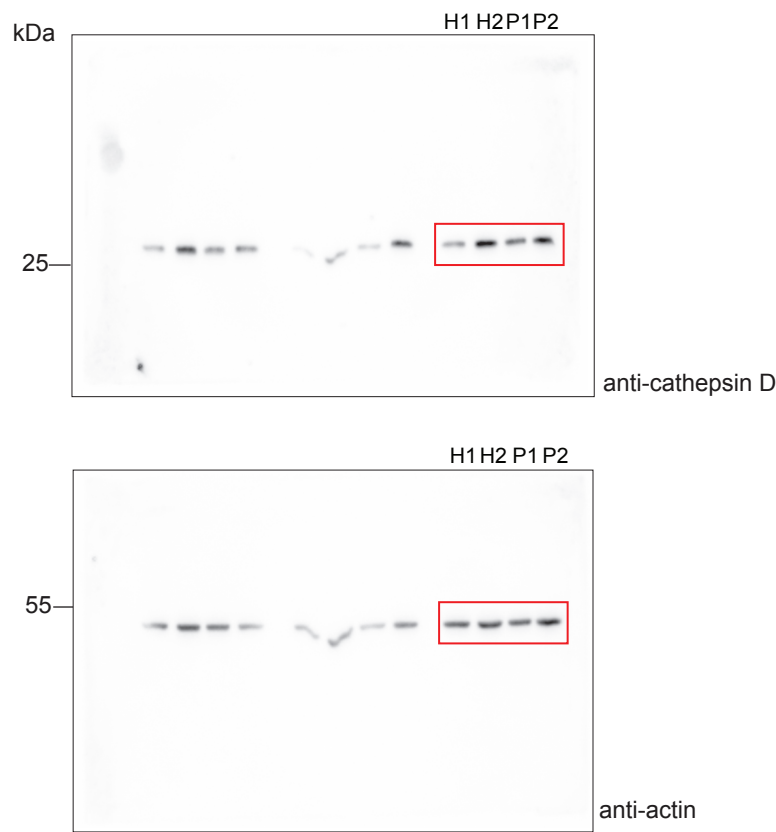

Supplement: SourceData F4 — is the source file for Fig. 4. [file JCB_202401167_SourceDataF4.pdf]

Source Data Figure7

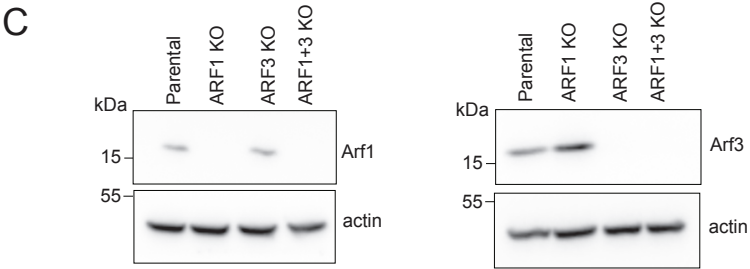

Source blots

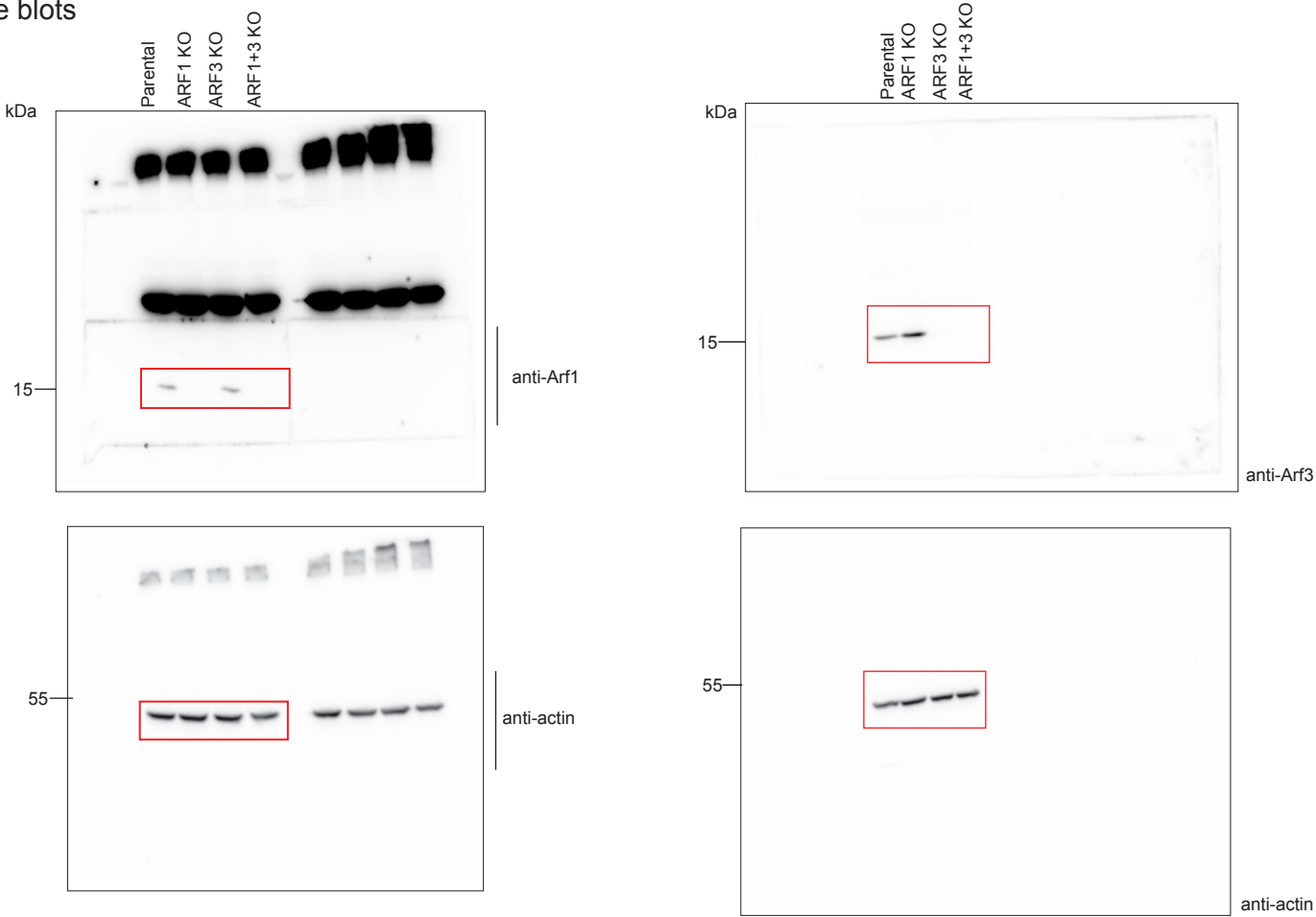

Supplement: SourceData F7 — is the source file for Fig. 7. [file JCB_202401167_SourceDataF7.pdf]

Source Data Figure 9

A

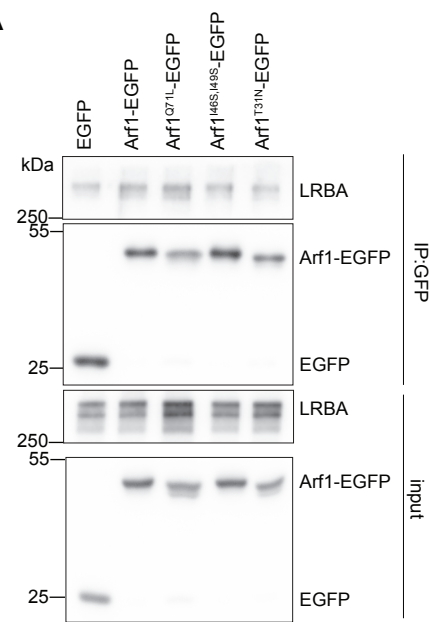

Source blots

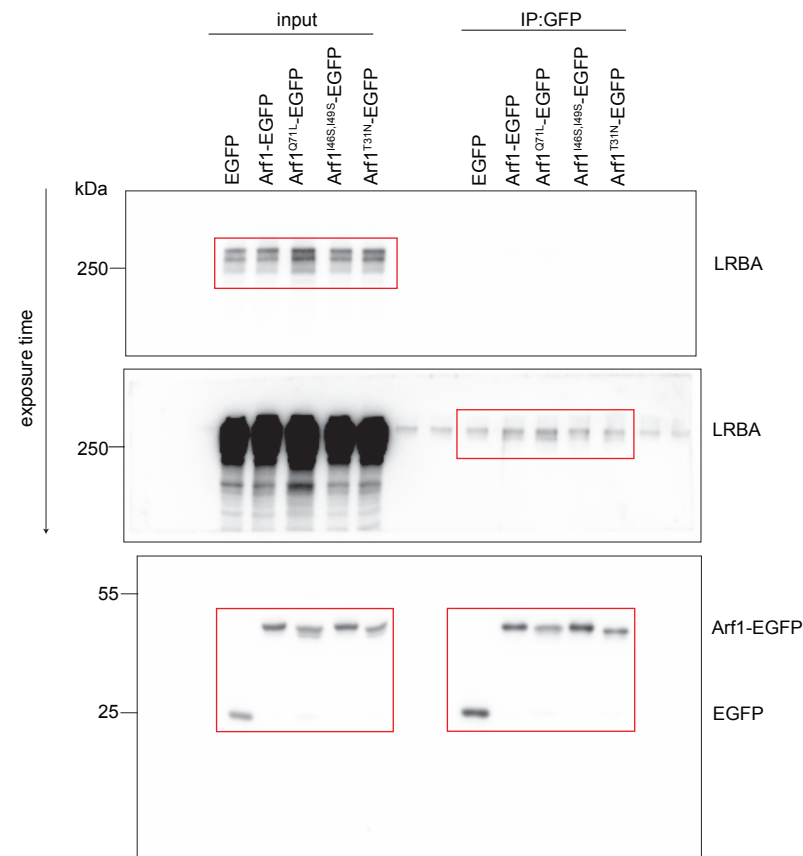

Supplement: SourceData F9 — is the source file for Fig. 9. [file JCB_202401167_SourceDataF9.pdf]

# Source Data Figure S2

E

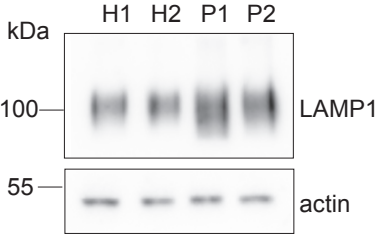

Source blots

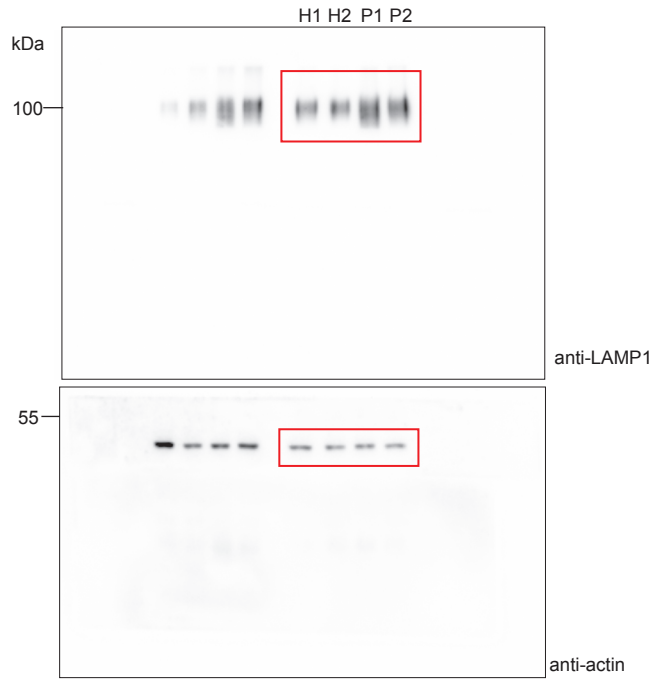

Supplement: SourceData FS2 — is the source file for Fig. S2. [file JCB_202401167_SourceDataFS2.pdf]
