# Supplementary material for: Arf1-dependent LRBA recruitment to Rab4 endosomes is required for endolysosome homeostasis
Source: J Cell Biol. 2024 Sep 26;223(11):e202401167. doi: 10.1083/jcb.202401167 (PMC11449124; doi:10.1083/jcb.202401167)

Source Data Figure S3

A

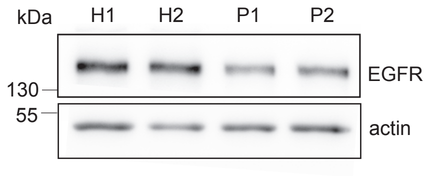

C

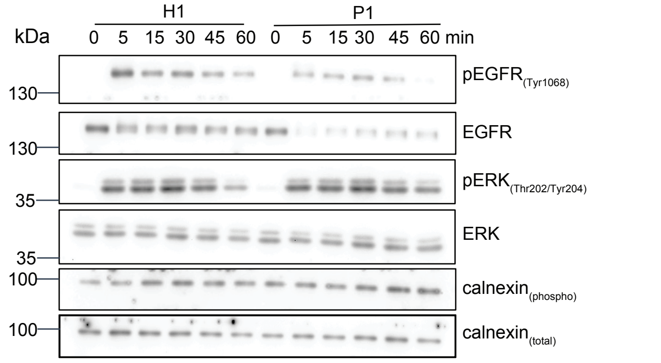

Source blots

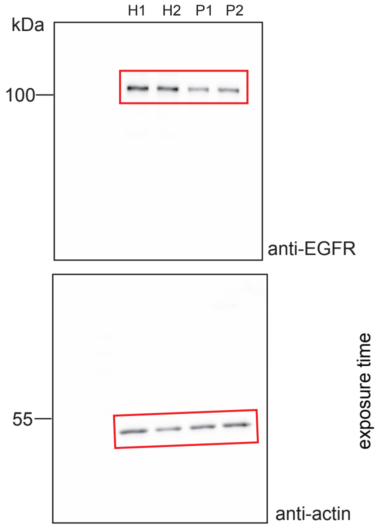

The same blot which is shown on Fig.1C has been stripped and re-probed with EGFR antibody.

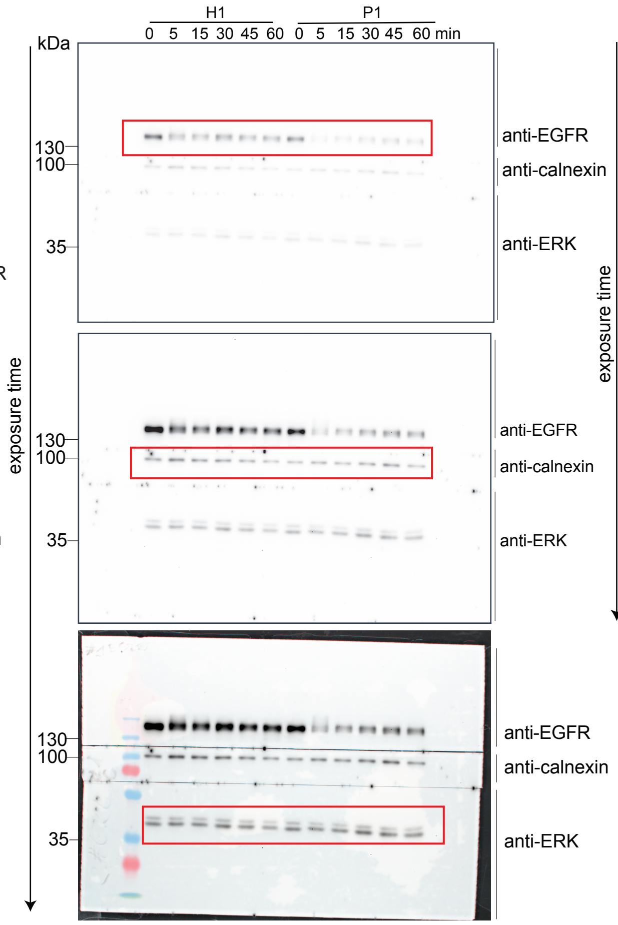

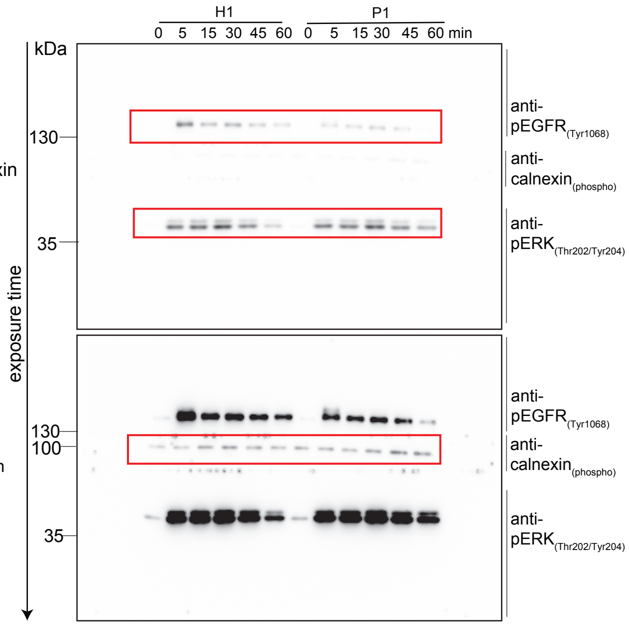

Supplement: SourceData FS3 — is the source file for Fig. S3. [file JCB_202401167_SourceDataFS3.pdf]
