# Supplementary material for: Arf1-dependent LRBA recruitment to Rab4 endosomes is required for endolysosome homeostasis
Source: J Cell Biol. 2024 Sep 26;223(11):e202401167. doi: 10.1083/jcb.202401167 (PMC11449124; doi:10.1083/jcb.202401167)
Supplement: SourceData FS5 — is the source file for Fig. S5. [file JCB_202401167_SourceDataFS5.pdf]

Source Data Figure S5

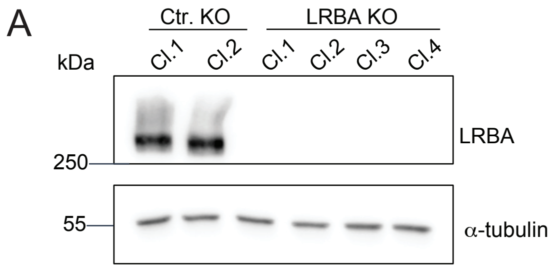

Source blots

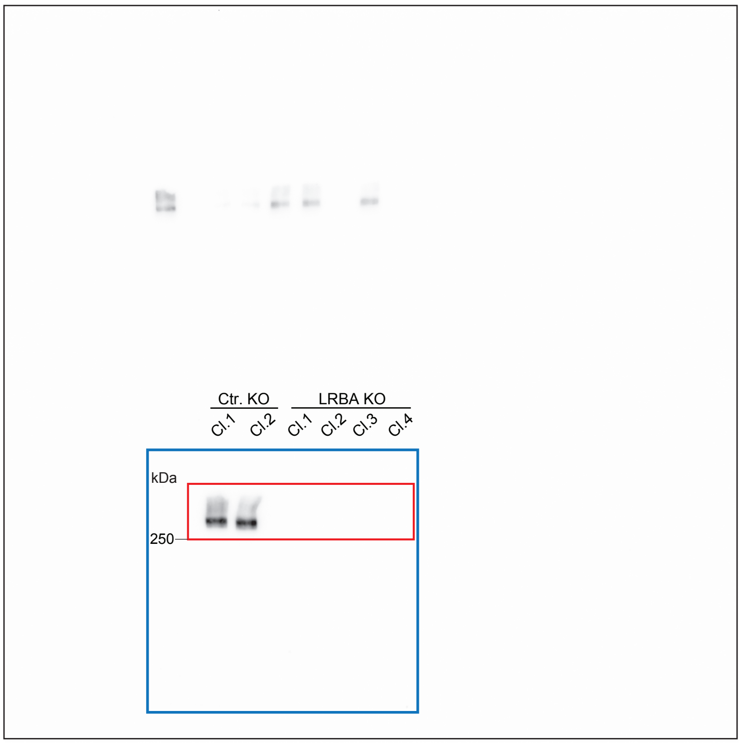

anti-LRBA

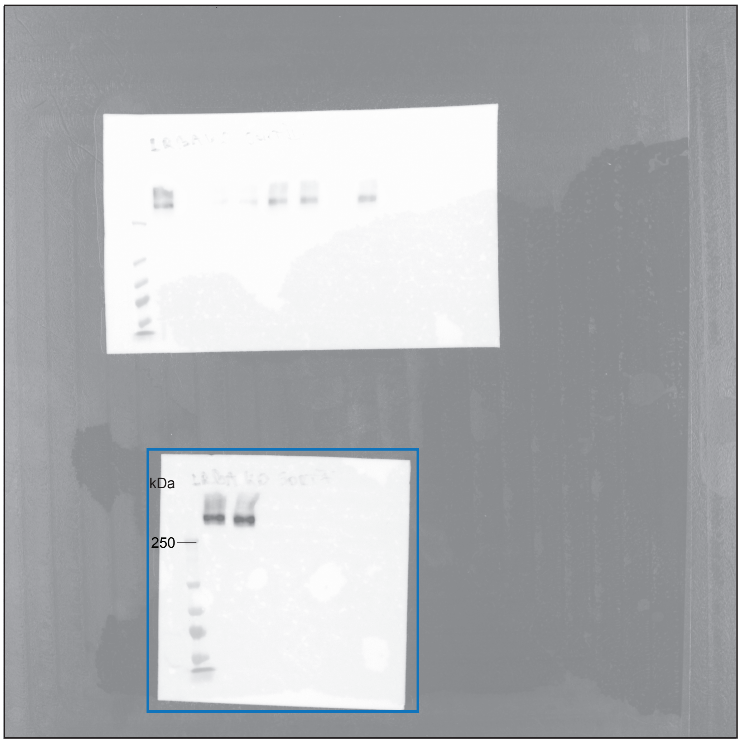

anti-LRBA+ladder

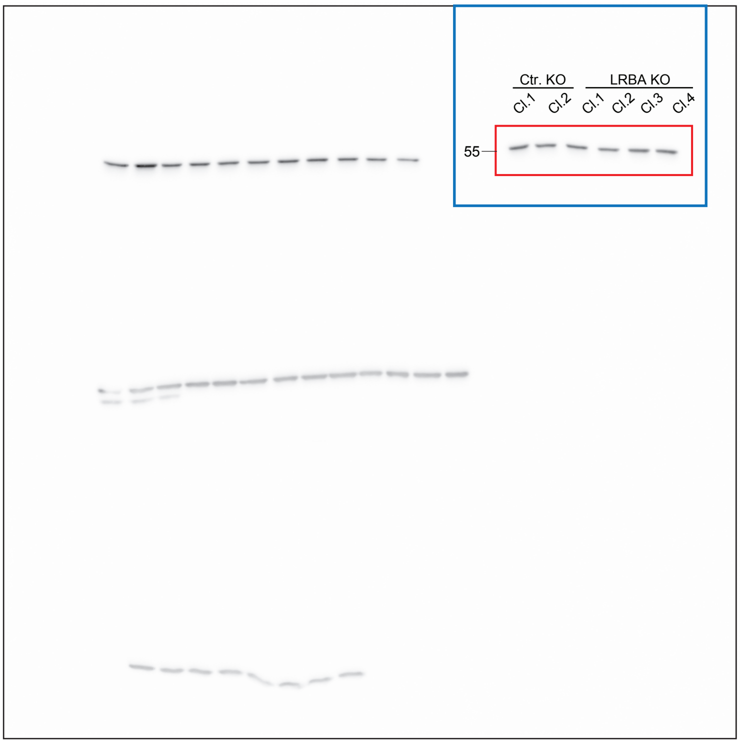

α–tubulin

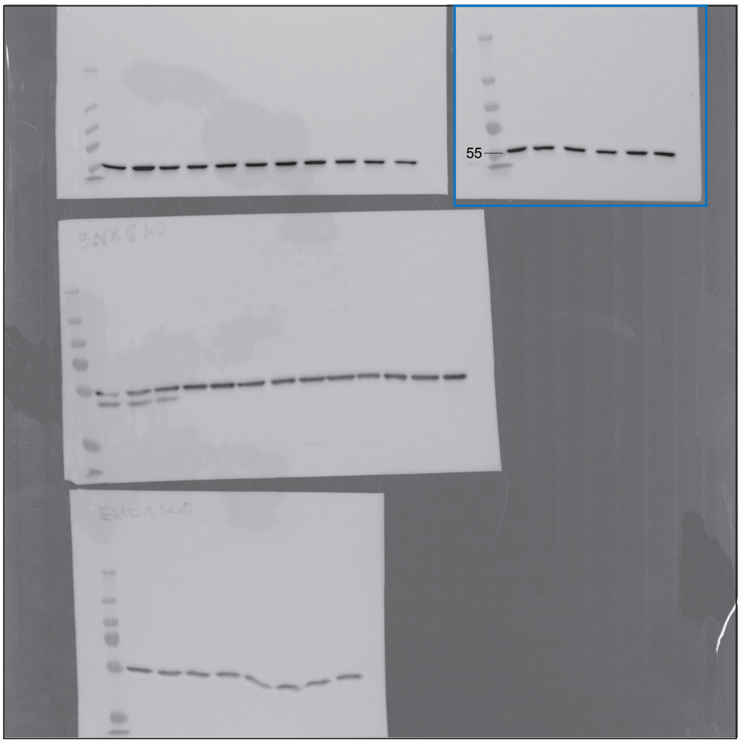

α–tubulin+ladder

cropped area shown on Figure S5, panel A

corresponding blot for Figure S5, panel A
